# Diagnostic Performance of Automated SARS-CoV-2 Antigen Assay in Nasal Swab during COVID-19 Vaccination Campaign

**DOI:** 10.3390/diagnostics11112110

**Published:** 2021-11-15

**Authors:** Haya Altawalah, Wadha Alfouzan, Talal Al-Fadalah, Sayeh Ezzikouri

**Affiliations:** 1 Department of Microbiology, Faculty of Medicine, Kuwait University, Safat 24923, Kuwait; alfouzan.w@ku.edu.kw; 2 Virology Unit, Yacoub Behbehani Center, Sabah Hospital, Ministry of Health, Safat 24923, Kuwait; 3 Laboratory Medicine, Farwania Hospital, Ministry of Health, Farwania 85000, Kuwait; 4 Qualities and Accreditation Directorate, Ministry of Health, Safat 13001, Kuwait; talfadalah@gmail.com; 5 Virology Unit, Viral Hepatitis Laboratory, Institut Pasteur du Maroc, Casablanca 20250, Morocco

**Keywords:** COVID-19, SARS-CoV2, Ag-RDT, diagnosis, rapid decisions

## Abstract

Background: To control the spread of the pandemic brought about by the severe acute respiratory syndrome coronavirus 2 (SARS-CoV-2) infection, it is necessary to have an automated reliable diagnostic assay. To date, the RT-PCR (RT-qPCR) has been the recommended laboratory method to diagnose SARS-CoV-2 infection, but there is a need for more automated and reliable tests. The aim of this real-life study was to assess the diagnostic performance of DiaSorin’s LIAISON SARS-CoV-2 antigen (Ag) chemiluminescence immunoassay in detecting SARS-CoV-2 in vaccinated and unvaccinated individuals. Methods: A prospective study was performed on 300 nasopharyngeal swabs randomly collected from 31 May to 6 July 2021. Nasopharyngeal samples were assayed with DiaSorin’s LIAISON SARS-CoV-2 Ag and TaqPath™ COVID-19 multiplex RT-qPCR. Results: Of 300 participants, 150 had a RT-qPCR confirmed SARS-CoV-2 infection of whom 113 (75.33%) were also detected by the DiaSorin LIAISON SARS-CoV-2 Ag. Taking RT-qPCR as a reference, the sensitivity and specificity of the DiaSorin LIAISON SARS-CoV-2 Ag assay were evaluated as 75.33% (95% CI = 67.64–82) and 100% (95% CI = 97.57–100), respectively. When a viral load cut-off was applied for high viral load (median cycle threshold (Ct) < 18.57), the overall sensitivity was increased to 96.55% (95% CI = 88.09–99.58). Interestingly, median RT-qPCR Ct and SARS-CoV-2 Ag values were similar between fully vaccinated and unvaccinated subjects. Conclusions: Automated, quantitative LIAISON SARS-CoV-2 Ag assay shows good performance to identify SARS-CoV-2-infected individuals with moderate to high viral loads. LIAISON SARS-CoV-2 Ag testing could be used as frontline testing for COVID-19 diagnosis and be more suitable for large utilization.

## 1. Introduction

Mass testing for the early identification and isolation of infectious coronavirus-19 disease (COVID-19) individuals is efficacious for reducing the disease spread [1]. In the ongoing pandemic context of COVID-19, diagnostic testing for SARS-CoV-2 is crucial in order to limit the spread of the virus as well as to appropriately manage infected patients [2]. To date, the gold standard test for severe acute respiratory syndrome coronavirus 2 (SARS-CoV-2) is the real-time RT-PCR (RT-qPCR) [3,4]. Moreover, RT-qPCR requires nasopharyngeal swabs (NPS) to be sent off to a special laboratory with specialist equipment, analyzed by competent laboratory staff and is time consuming (4–6 h), not including the time to transport the specimens to the laboratory [3]. Simpler and less expensive antigen-based tests have been developed to address these issues. However, antigen-detecting rapid diagnostic tests (Ag-RDT) require a visual readout and lack proper internal quality control, making them more prone to errors [5,6,7]. However, a number of automated antigen-detecting diagnostic assays for SARS-CoV-2 detection are now commercially available and can result in rapid decisions on patient care, isolation and contact tracing at the point of care [8]. As a part of the surveillance program for pandemic control in Kuwait, the local government launched nucleic acid amplification test-based systematic screenings and several other tools in the country in order to rapidly mitigate and manage COVID-19 patients [9].

To date, comparative studies have almost been completed for Ag-RDT with RT-qPCR testing and have shown high reliability before the vaccination campaign against COVID-19 [8,10,11,12]. COVID-19 vaccines reduce severe disease and death from SARS-CoV-2 infection [13,14] but breakthrough cases occur [15,16]. Several reports have shown no difference in terms of viral loads between vaccinated and unvaccinated subjects. However, other studies have found that COVID-19 vaccines reduce viral loads [17,18]. This situation emphasizes the importance to replicate other investigations in other populations. Moreover, the general application of antigen testing deserves to be further investigated and validated on larger cohorts, taking into account also the current spreading of the novel more infectious SARS-CoV-2 variants [19,20]. The SARS-CoV-2 variants may present important diagnostic challenges in the future. The rapid implementation of a COVID-19 antigen assay requires critical assessment. Thus, the objective of this real-life study was to evaluate a quantitative antigen assay in detecting SARS-CoV-2 in vaccinated and unvaccinated individuals. The first part of the study was to evaluate the performance of the DiaSorin LIAISON SARS-CoV-2 Ag test in detecting SARS-CoV-2 compared to RT-qPCR. The second part of the study was to assess the limit of detection between SARS-CoV-2 Ag and RT-qPCR assays. The third part of the investigation was to analyze the viral loads between vaccinated and unvaccinated subjects.

## 2. Materials and Methods

### 2.1. Clinical Specimens

To collect nasopharyngeal swab specimens (NPS), the swab was passed through the nostril until reaching the posterior nasopharynx and removed while rotating as previously described [9]. NPS were collected by health-care professionals from individuals at the AnaSalbi laboratory, a certified COVID-19 testing laboratory in Kuwait. After swabbing, each absorbent swab was placed immediately into a sterile tube with copan universal transport media (UTM). We conducted a cross-sectional study from 31 May to 6 July 2021, with a nested sampling from positive and negative samples. One hundred and fifty negative and 150 positive specimens were randomly sampled from the different NPS stored in the laboratory. Metadata including demographic data, vaccination status, asymptomatic, symptomatic were collected. The study protocol was approved by the permanent Committee for Coordination of Medical and Health Research, Ministry of Health, Kuwait, and the study was conducted in accordance with the ethical guidelines of the 1975 Declaration of Helsinki as reflected in a priori approval by the institution’s human research committee.

### 2.2. SARS-CoV-2 RT-q PCR

Viral RNA was automatically extracted from 200 μL of the NPS specimens using the MagMAX^TM^ Viral/Pathogen II Nucleic Acid Isolation Kit (Thermo Fisher Scientific, Vilnius, Lithuania) on KingFisher (Thermo Fisher Scientific, Waltham, MA, USA) according to the manufacturer’s instructions. RT-qPCR was performed using TaqPath™ COVID-19 multiplex real-time RT-PCR test (the Orf1ab, N and S genes) (Thermo Fisher Scientific, Waltham, MA, USA) according to the manufacturer’s instructions. Negative and positive controls were run simultaneously with samples [2]. The assay was performed using a QuantStudio 5 real-time PCR detection system (Thermo Fisher Scientific, Waltham, MA, USA). Test results were reported quantitatively as cycle threshold (Ct) value of the ORF1ab, the N and the S genes, while qualitative data were reported as positive/negative at test cut-off (i.e., Ct < 37 for positive or ≥ 37 for negative samples). The Ct values used to classify specimens as ‘high viral load’, ‘medium viral load’ and ‘low viral load’ defined as previously described [7,21], they were <18.57, 18.57–28.67 and >28.67, respectively.

### 2.3. LIAISON SARS-CoV-2 Ag

The DiaSorin LIAISON SARS-CoV-2 Ag assay is a two-step sandwich fully automated chemiluminescence immunoassay (CLIA) for the quantitative determination of SARS-CoV-2 nucleocapsid (N) antigen protein in nasopharyngeal swabs on the LIAISON^®^XL Analyzer (DiaSorin, Saluggia, Italy). The assay was performed according to the manufacturer’s recommendations. The light signal, and hence the amount of isoluminol-antibody conjugate, is measured by a photomultiplier in relative light units (RLU) (TCID_50_/mL) and indicates the presence or absence of the SARS-CoV-2 Ag in samples.

### 2.4. Statistical Analysis

RT-qPCR was considered as the gold standard for this evaluation, therefore, positive and negative samples by molecular techniques were considered to be true positive and true negative samples, respectively. Sensitivity, specificity, positive and negative predictive values with corresponding 95% confidence intervals (CI) were calculated to assess diagnostic performance. Student *t* and Mann–Whitney U tests were used to assess differences between groups. Categorical variable was compared using Chi-square test. The area under the receiver operating characteristics curves (AUC of ROC) and their 95% confidence intervals (CIs), were used to evaluate the diagnostic value of the LIAISON SARS-CoV-2 Ag assay. A simple linear regression was performed to assess the potential correlation between antigen level obtained on the automated LIAISON^®^ SARS-CoV-2 antigen test and RT-qPCR Ct values. All *p*-values were two-sided and *p* less than 0.05 was considered significant. Statistical analyses were performed using GraphPad PRISM version 6.0e (GraphPad Software, San Diego, CA, USA) or MedCalc statistical software.

## 3. Results

### 3.1. Sensitivity and Specificity of the Automated LIAISON^®^ SARS-CoV-2 Antigen Test

We randomly sampled 300 nasopharyngeal specimens. According to RT-qPCR results, 150 specimens were positive collected from asymptomatic and symptomatic patients and 150 were negative. The demographic and clinical characteristics of the study subjects are shown in Table 1. Vaccinated persons received two doses of Pfizer–BioNTech’s messenger RNA-based vaccine (BNT162b2), or ChAdOx1 nCoV-19 vaccine, Oxford/AstraZeneca’s non-replicating viral-vectored vaccine. There was no significant difference between the proportions of vaccinated people (53.3%) with symptoms and unvaccinated patients (46.7%) (*p* = 0.766).

Among the 150 positive samples by RT-qPCR, 113 were positive for SARS-CoV-2 Ag according to the manufacturer interpretation rules (<100 TCID_50_/mL for negative, 100–199.99 TCID_50_/mL for equivocal, and ≥200 TCID_50_/mL for positive sample). The diagnostic accuracy of COVID-19 using SARS-CoV-2 Ag was performed (Table 2). Using NPS RT-qPCR as the reference method, the sensitivity of SARS-CoV-2 Ag for the diagnosis of COVID-19 in nasopharyngeal swabs was 75.33% (95% CI: 67.64–82). Among the 39 false negative samples, ten were located in the 100–199.99 TCID_50_/mL range defined by the manufacturer as equivocal, while 29 were negative (<100 TCID_50_/mL). Among the 150 negative samples by RT-qPCR, 150 were negative for SARS-CoV-2 antigen detection, meaning an overall specificity of 100% (95% CI: 97.57–100). A deep assessment was performed and summarized in Table 2.

**Figure 1:**
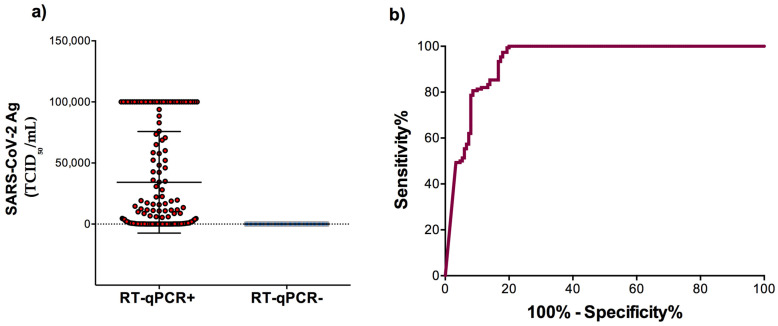
SARS-CoV-2 antigen detection according to RT-qPCR results. (**a**) The level of SARS-CoV-2 Ag among the RT-qPCR positive and negative samples. (**b**) Receiver operating characteristic (ROC) curve analysis to evaluate the diagnostic value of the LIAISON SARS-CoV-2 Ag assay. Data are presented as mean ± SD.

The mean level of SARS-CoV-2 Ag among the RT-qPCR positive samples was significantly higher (34,202 ± 3395 TCID_50_/mL) than that of the RT-qPCR negative samples (41.44 ± 2.08 TCID_50_/mL) (*p*< 0.0001) (Figure 1a). ROC curve analysis was performed to determine the area under the curve (AUC) of the antigen level allowing the distinction of SARS-CoV-2 infection status. The AUC of the assay was 0.94 (95% CI, 0.91–0.96; *p* < 0.0001) indicating a very good performance (Figure 1b).

### 3.2. Antigen Detection According to the RT-qPCR Ct Values

**Figure 2:**
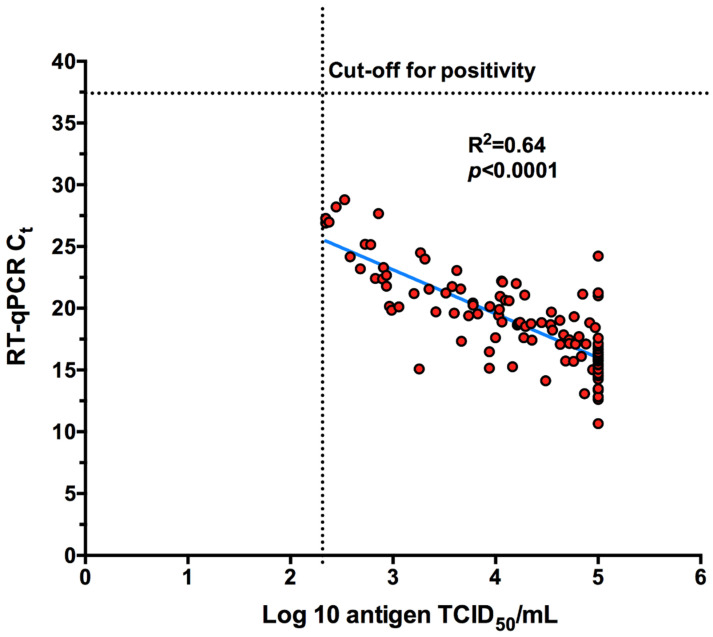
Linear regression of RT-qPCR Ct values versus the level of antigen (log10 transformed TCID50/mL results) obtained on the DiaSorin LIAISON SARS-CoV-2 Ag assay.

Data analysis showed a good correlation between the antigen level and RT-qPCR Ct values (Figure 2).

The sensitivity was evaluated according to the Ct value. The viral load cut-off was applied to classify specimens as high viral load (Ct < 18.57), medium viral load (18.57 < Ct < 28.67) and low viral load (Ct > 28.67) (based on the average Ct SARS-CoV-2 genes) (Table 3). The sensitivity of the SARS-CoV-2 Ag detection was 96.55% when considering samples with high viral load. The median level of SARS-CoV-2 Ag was 100,000 TCID_50_/mL (range 1800–100,000 TCID_50_/mL). However, the sensitivity of the DiaSorin LIAISON SARS-CoV-2 Ag assay dropped to 6.67% for samples with low viral load (Table 3).

### 3.3. RT-qPCR, Antigen Detection and COVID-19 Vaccination

**Figure 3:**
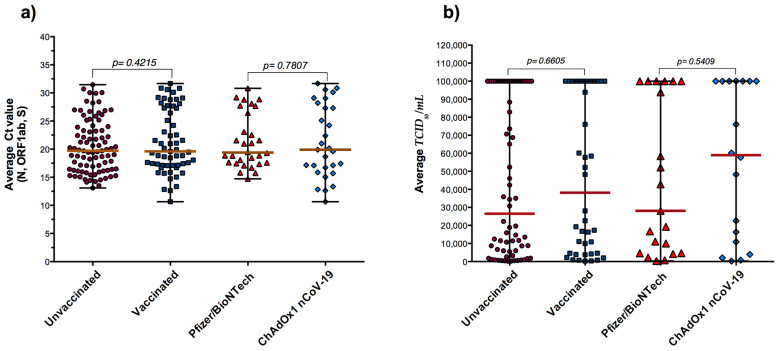
Cycle threshold (Ct) values for PCR-positive infections by vaccination status and type of vaccine. (**a**) Data are presented as RT-qPCR Ct values. (**b**) Data are presented as SARS-CoV-2 antigen levels obtained on the DiaSorin LIAISON SARS-CoV-2 Ag assay. Data are presented as median with range.

The samples were classified according to the COVID-19 vaccination status (Table 1). The Ct values were analyzed in fully vaccinated and unvaccinated subjects according to viral loads (Figure 3a) and antigen quantification comparisons (Figure 3b). Interestingly, fully vaccinated individuals had relatively similar viral loads (Median PCR Ct = 19.60) as unvaccinated individuals (Median PCR Ct = 19.70) (*p* = 0.4215) (Figure 3a). Notably, the 113 positive subjects using automated LIAISON^®^ SARS-CoV-2 antigen assay (TCID_50_/mL ≥ 200) were stratified according to their vaccination status and data revealed no significant difference between fully vaccinated and unvaccinated subjects regarding the level of SARS-CoV-2 Ag values (Figure 3b). In addition, stratification according to vaccine type showed no significant differences between Pfizer/BioNTech and ChAdOx1 nCoV-19 vaccines (Figure 3).

## 4. Discussion

Viral nucleic acid detection using an RT-qPCR assay remains the standard diagnosis tool of COVID-19. While this detects a large number of the suspected and contact cases with typical clinical COVID-19 features, other alternative diagnostic approaches are needed. The SARS-CoV-2 antigen diagnostic assays have been widely used to help diagnosis virus infection. Compared to the RT-qPCR assay, the automated LIAISON SARS-CoV-2 Ag is often faster (around 42 min), less expensive, easier to use and accessible to staff without laboratory training. In addition, the detection ability of the N antigen by the DiaSorin LIAISON SARS-CoV-2 Ag is not theoretically impacted by the recently emerged variants of concern, which harbor different mutations in the spike protein [22,23]. Here, we evaluated the performance characteristics of the DiaSorin LIAISON SARS-CoV-2 Ag assay for detecting SARS-CoV-2 in respiratory specimens and compared the results with RT-qPCR (the gold standard assay). The sensitivity and specificity of this assay for the detection of SARS-CoV-2 were presented and the clinical application of this assay for the diagnosis of COVID-19 was discussed.

The overall sensitivity of DiaSorin LIAISON SARS-CoV-2 Ag assay was evaluated to be 75.33% and the specificity reached 100%. Previous investigations of the LIAISON SARS-CoV-2 Ag test showed an overall sensitivity ranging from 31 to 84.8% and specificity of ≈100% at the 200 TCID_50_/mL manufacturer’s cut-off [5,24,25,26], whereas, when applying the cut-off at 82 TCID_50_/mL, a previous report showed that the sensitivity of the DiaSorin LIAISON SARS-CoV-2 Ag test yielded 73% sensitivity [26]. Moreover, similar automated antigenic assays have previously been evaluated and exhibited sensitivity ranging from 70% to 97% [21,22,27,28,29,30,31,32,33].

In this study, the diagnostic performance of the novel DiaSorin LIAISON SARS-CoV-2 Ag chemiluminescence immunoassay suggests a higher performance in samples collected in the early phase of infection with high viral loads (i.e., Ct values Ct < 18.57) and displaying 96.55% sensitivity. These data seem to be in line with previous studies [7,21,24,26,31,34].

A major finding in our study is a comparison of Ct values and LIAISON SARS-CoV-2 Ag levels highlighting no significant differences between vaccinated and unvaccinated individuals in term of viral RNA loads and SARS-CoV-2 Ag levels, suggesting the same transmission potential. These data seem to be in line with several previous investigations showing no significant differences between vaccinated and unvaccinated groups, with significant breakthrough infections of SARS-CoV-2 variants in fully vaccinated people [33,35,36,37,38,39,40,41]. However, previous studies showed that COVID-19 vaccines attenuated the SARS-CoV-2 RNA loads, indicating a reduced infectiousness and virus transmissibility [17,18,42].

## 5. Conclusions

This study showed that the automated DiaSorin LIAISON SARS-CoV-2 antigen test has good sensitivity and specificity for identifying patients with high SARS-CoV-2 viral load and at high risk of being active sources of contagion. However, the sensitivity level does not allow for exclusion of the risk that subjects testing negative may still carry the active SARS-CoV-2 virus. Thus, more investigations are warranted by companies to improve its sensitivity in order to reduce false negative results regarding samples with low viral loads. Notably, no significant differences were observed between vaccinated and unvaccinated subjects in terms of viral loads and SARS-CoV-2 Ag levels. Thus, booster vaccinations in groups at high risk of severe COVID-19 should be recommended to help reduce the burden of COVID-19 and to improve vaccination responses.

In summary, DiaSorin’s LIAISON SARS-CoV-2 antigen is an inexpensive assay and an ideal test for high-throughput and decentralized screening of subjects with high SARS-CoV-2 viral loads for the early identification and isolation of COVID-19 patients.

## Figures and Tables

**Table 1:** Demographic and clinical characteristics of subjects.

	RT-qPCR Positive Patients (N = 150)	RT-qPCR Negative Subjects (N = 150)
Median age (range), year	34 (4–74)	43 (7–79)
Sex, no. (%)		
Male	54 (36)	84 (56)
Female	96 (64)	66 (44)
Presenting symptoms and signs, no. (%)		
Fever	12 (8)	6 (4)
Headache	10 (6.7)	1 (0.7)
Cough	14 (9.3)	4 (2.7)
Generalised weakness	12 (8)	3 (2)
Nasal congestion	9 (6)	-
Sore throat	5 (3.3)	2 (1.3)
Ageusia/Anosmia	10 (6.7)	3 (2)
Diarrhea	3 (2)	2 (1.3)
Shortness of breath	7 (4.7)	2 (1.3)
COVID-19 vaccine, no. (%)		
Pfizer–BioNTech	30 (20)	44 (29.3)
ChAdOx1 nCoV-19	29 (19.3)	47 (31.3)

**Table 2:** Assessment of the diagnostic accuracy of the automated LIAISON^®^ SARS-CoV-2 antigen test in nasopharyngeal swabs.

	Value (%)	(95% CI)
Sensitivity	75.33	(67.64–82.00)
Specificity	100.00	(97.57–100.00)
Negative likelihood ratio	0.25	(0.19–0.3)
Positive predictive value	100	(92.32–99.13)
Negative predictive value	80.21	(75.40–84.28)
Accuracy	87.67	(83.40–91.17)

**Table 3:** Performance of the diagnostic accuracy of antigen detection according to the RT-qPCR Ct values.

SARS-CoV-2 Antigen Detection					
Viral Load	Median Ct Value (Range)	*n*	Positive	Median SARS-CoV-2 Ag Value TCID_50_/mL (Range)	Sensitivity
High	15.94 (10.65–18.53)	58	56	100,000 (1800–100,000)	96.55%
Medium	22.29 (18.64–28.52)	77	56	5736 (220–100,000)	72.73%
Low	30.07 (28.79–31.67)	15	1	336	6.67%
All	19.90 (10.65–31.67)	150	113	25,353 (220–100,000)	75.33%

## Data Availability

Data supporting reported results are available from the corresponding author on reasonable request.
